# Function and Regulation of Nuclear DNA Sensors During Viral Infection and Tumorigenesis

**DOI:** 10.3389/fimmu.2020.624556

**Published:** 2021-01-11

**Authors:** Fan Zhang, Yi Yuan, Feng Ma

**Affiliations:** ^1^ Key Laboratory of Synthetic Biology Regulatory Elements, Chinese Academy of Medical Sciences & Peking Union Medical College, Beijing, China; ^2^ Suzhou Institute of Systems Medicine, Suzhou, China; ^3^ Department of Laboratory Medicine, Shanghai Tongji Hospital, School of Medicine of Tongji University, Shanghai, China

**Keywords:** nuclear DNA sensor, IFI16, hnRNPA2B1, cGAS, p53, type I interferon, tumorigenesis

## Abstract

IFI16, hnRNPA2B1, and nuclear cGAS are nuclear-located DNA sensors that play important roles in initiating host antiviral immunity and modulating tumorigenesis. IFI16 triggers innate antiviral immunity, inflammasome, and suppresses tumorigenesis by recognizing double-stranded DNA (dsDNA), single-stranded DNA (ssDNA), damaged nuclear DNA, or cooperatively interacting with multiple tumor suppressors such as p53 and BRCA1. hnRNPA2B1 initiates interferon (IFN)-α/β production and enhances STING-dependent cytosolic antiviral signaling by directly binding viral dsDNA from invaded viruses and facilitating *N^6^*-methyladenosine (m^6^A) modification of cGAS, IFI16, and STING mRNAs. Nuclear cGAS is recruited to double-stranded breaks (DSBs), suppresses DNA repair, and promotes tumorigenesis. This review briefly describes the nuclear functions of IFI16, hnRNPA2B1, and cGAS, and summarizes the transcriptional, post-transcriptional, and post-translational regulation of these nuclear DNA sensors.

## Introduction

The first line of host defense against pathogenic threats is orchestrated by the innate immune system, which relies on the ability of immune cells to recognize the presence of extracellular or intracellular pathogen-associated molecular patterns (PAMPs) through germline-encoded pattern recognition receptors (PRRs) (1). Viral nucleic acids are the main PAMPs generated during viral infection. Once infected, the interactions between PRRs and viral nucleic acids evoke a series of signaling transduction cascades that lead to the initiation of cell defense to eliminate viruses. For instance, recognition of viral DNA by cytosolic DNA sensors like cyclic GMP-AMP synthase (cGAS) elicits the activation of the adaptor protein stimulator of interferon genes (STING), which further recruits and activates TANK-binding kinase 1 (TBK1) and interferon-regulatory factor 3 (IRF3) (2–4). STING also activates the transcription factor nuclear factor-κB (NF-κB), which subsequently collaborates with IRF3 to promote the expression of type I IFNs (IFN-Is) and proinflammatory cytokines (5–7). Additionally, cytosolic DNA binds to the receptor absent in melanoma 2 (AIM2), leading to the recruitment of the apoptosis-associated speck-like protein containing CARD (ASC) and pro-caspase-1 to assemble a multi-protein complex termed inflammasome, which constitutes a group of PRRs and plays essential roles in response to viral infection (8). Once assembled, the AIM2 inflammasome complex further promotes the proteolytic maturation and secretion of proinflammatory cytokines, including interleukin 1 beta (IL-1β) and IL-18, thereby initiating the inflammatory cascade (9).

Although the stimulation of cytosolic nucleic acid sensors by viral nucleic acids is critical for host antiviral defense, multiple viruses replicate in the nucleus with much less or no opportunities for cytosolic engagement of viral nucleic acids. In the past few years, accumulating evidence has demonstrated that nuclear DNA sensors, such as IFN-γ-inducible protein 16 (IFI16), heterogeneous nuclear ribonucleoprotein A2/B1 (hnRNPA2B1), and nuclear cGAS, also exert critical roles in initiating host antiviral immunity (10). However, compared to the wealth of knowledge about cytosolic DNA sensors and the other PRRs, studies for the roles and underlying mechanisms of nuclear DNA sensors are only just emerging (11–13). Recent evidence indicates that nuclear DNA sensors are also involved in tumor development beyond pathogenic DNA recognition. Aberrant or damaged self-DNA species generated due to genomic instability serve as ligands to engage these nuclear DNA sensors during tumorigenesis.

This review focuses on the latest findings to provide a more comprehensive understanding of the functions of nuclear DNA sensors during viral infection and tumorigenesis. It also summarizes the regulation of these nuclear DNA sensors, including transcriptional, post-transcriptional, and post-translational regulation during viral infection and tumorigenesis.

## Nuclear DNA Sensors Facilitate Antiviral Immunity 

IFI16 is a member of the pyrin and HIN200 domain-containing protein family (PYHIN) that contains a pyrin domain and two DNA-binding HIN domains. It has been identified as a nuclear DNA sensor that mediates the induction of IFN-Is (14). Upon detecting viral DNA in the nucleus, IFI16 translocates to the cytoplasm where it oligomerizes and relays signals through adaptor molecule STING, engaging the TBK1-IRF3 axis and the NF-κB pathway to induce the transcription of IFN-Is (2, 15, 16). IFI16 has also been shown to interact with Kaposi’s sarcoma-associated herpesvirus (KSHV) genomic DNA in the nucleus, leading to the formation of a functional inflammasome. Different from the cytosolic AIM2 inflammasome, the IFI16 inflammasome complex is initially assembled in the nucleus and subsequently translocates to the cytoplasm, suggesting a nucleus-associated inflammasome sensor component against KSHV infection (17, 18). The overexpression of IFI16 with other inflammasome components in HEK293T cells is of note as it exhibits a low-level production of IL-1β. When these cells are infected by KSHV, an elevated level of IL-1β is observed, implying that the IFI16 inflammasome requires additional cofactors for optimal activation. The work of Brunette et al. further supports this notion that IFI16 and its mouse homolog p204 are poor activators of either STING-dependent IFNs or ASC-inflammasome, while AIM2 robustly activates both IFNs and the inflammasome in an experimental overexpression system (19).

hnRNPA2B1 is a member of the hnRNP family and has been recently identified as a nuclear DNA sensor (12). Upon sensing viral DNA in the nucleus, hnRNPA2B1 dimerizes and is demethylated by arginine demethylase JMJD6, which results in the cytoplasmic translocation of hnRNPA2B1. The cytoplasmic hnRNPA2B1 dimers interact with STING and activate the TBK1-IRF3 signal transduction cascade to facilitate the transcription of downstream IFN-Is. Moreover, hnRNPA2B1 can disassociate with fat mass and obesity-associated protein (FTO) after virus infection, leading to the promotion of *N^6^*-methyladenosine (m^6^A) modification, nucleocytoplasmic trafficking, and translation of cGAS, STING, and IFI16 mRNAs to amplify the activation of IFN-Is in antiviral innate immune response (20). A recent study shows that hnRNPA2B1 plays a vital role in transporting herpes simplex virus 1 (HSV-1) from the envelopment site to the extracellular environment (21). Interestingly, hnRNPA2B1 facilitates the replication of hepatitis E virus (HEV), an ssRNA virus, though hnRNPA2B1 is initially identified as a DNA sensor (22).

cGAS is a member of the nucleotidyltransferase family, the binding of cytoplasmic pathogenic DNA to cGAS induces a phase transition to liquid-like droplets, promoting the production of the secondary messenger cyclic guanosine monophosphate–adenosine monophosphate (cGAMP) and subsequent induction of IFN-Is through the STING-TBK1-IRF3 signaling axis (23, 24). cGAS mainly localizes in the cytoplasm, yet cGAS expresses in interphase and may translocate to the nucleus due to nuclear envelope rupture or mitosis (24, 25). Nuclear cGAS usually maintains a suppressed state by chromatin tethering to limit reactivity against self-DNA (26, 27). A recent study reveals that upon nuclear entry of the human immunodeficiency virus (HIV), NONO, an innate immune sensor of the viral capsid proteins is associated with cGAS in the nucleus and is required to retain cGAS in the nucleus but has no impact on the cytosolic pool of cGAS. The crosstalk between NONO and cGAS in the nucleus enables the sensing of DNA intermediate during HIV infection. The detection of the nuclear viral capsid by NONO promotes DNA sensing by cGAS and reveals an innate strategy of distinguishing viruses from self in the nucleus (28).

## IFI16 Suppresses Viral Replication as a Transcriptional Repressor 

Several studies have reported that IFI16 functions as a transcriptional repressor (29). For instance, IFI16 has been described as a restriction factor for human cytomegalovirus (HCMV) replication on account of suppressing the transcriptional activity of the viral DNA polymerase gene (UL54) (30). Besides, IFI16 transcriptionally represses HSV-1 gene expression such as the immediate-early proteins (ICP0 and ICP4), the early proteins (ICP8 and TK), and the late proteins (GB and Us11), and limits viral replicative capacity (31, 32). IFI16 has also been demonstrated to function as a restriction factor for human papillomavirus 18 (HPV18) replication through histone modifications (33). A recent study shows that IFI16 limits HIV-1 transcription and latency reactivation by targeting the transcription factor Sp1 (34). Overall, these data identify IFI16 as a transcriptional repressor for various DNA viruses in the nucleus, of which the mechanisms still need deeper investigation.

Most studies suggest that IFI16 modulates transcription mainly through association with transcription factors or promoters. As mentioned above, IFI16 binds to the transcription factor Sp1 to suppress HIV-1 transcription (34). Similarly, Cristea et al. show that IFI16 interacts with the major immediate-early promoter (MIEP), and participates in controlling the viral immediate-early gene transcription by HCMV virion protein pUL83 (35). In addition to associating with transcription factors or promoters directly, IFI16 prevents transcription factors from interacting with their promoters. For instance, IFI16 has been shown to inhibit the association of some transcription factors such as Sp1 with the HCMV promoter (30).

Additionally, a study shows that IFI16 blocks the interaction of transcription factors, TATA-binding protein (TBP), and Octamer-Binding Transcription Factor 1 (Oct 1), with HSV-1 promoters (31). Meanwhile, the study also suggests that IFI16 may facilitate global histone modifications by modulating the formation of heterochromatin and euchromatin for both viral and cellular genes. Consequently, IFI16 may modulate transcription through chromatin modification. Another study also suggests that IFI16 promotes the addition of heterochromatin marks and the reduction of euchromatin marks on viral chromatin, thereby inhibiting viral gene expression and replication (36). Furthermore, IFI16 promotes the assembly of heterochromatin on HPV DNA, thus reducing both viral replication and transcription (33). Altogether, IFI16 is involved in transcriptional repression through association with transcription factors or promoters, preventing transcription factors from binding to their promoters and inducing changes in chromatin markers.

## Nuclear DNA Sensors Regulate Tumorigenesis

Despite the essential roles of nuclear DNA sensors in the host antiviral defense, studies on these PRRs have also been well documented in the absence of infection. IFI16 acts as a DNA damage amplifier by interacting with p53 through its C-terminal domain and consequently promotes the accumulation and activation of p53 caused by DNA damage (37, 38). Increased levels of IFI16 promote the transcription of known p53 target genes, such as the cell cycle kinase inhibitor p21 and the proapoptotic Bcl-2 family member Bax, inducing p53-mediated cell cycle arrest and apoptosis in human cancer cells (38, 39). Decreased IFI16 mRNA expression is observed in numerous breast cell lines, which results in dysfunction of p53-mediated apoptosis and leads to cancer development (38). Subsequently, Lin et al. show that IFI16 functions as a tumor suppressor in hepatocellular carcinoma (HCC) by activating the p53 signaling pathway and inflammasome (40). In turn, functional activation of p53 stimulates the transcription of IFI16 through associating with the regulatory region of the IFI16 gene in the cells treated with DNA-damaging agents, suggesting a positive feedback loop between p53 and IFI16 (41). A recent research indicates that IFI16 positively regulates programmed cell death 1 ligand 1 (PD-L1) in cervical cancer cells by activating the STING-TBK1-NF-κB pathway, which can interact with the proximal region of the PD-L1 promoter to facilitate PD-L1 expression, and promoting the progression of cervical cancer (42).

Studies have also provided evidence that hnRNPA2B1 functions as a putative proto-oncogene in some cancers such as glioblastoma, pancreatic cancer, liver cancer, and pancreatic ductal adenocarcinoma (PDAC) (43–46). The upregulated expression of hnRNPA2B1 facilitates the malignant phenotypes of cancer cells by modulating many downstream target genes. hnRNPA2B1 is also overexpressed in a variety of other tumors. For instance, the expression of hnRNPA2B1 in human ovarian cancer tissues is significantly higher than that in normal ovarian epithelium tissues, and increased hnRNPA2B1 level is related to the poor prognosis of ovarian cancer patients (47). hnRNPA2B1 also serves as a diagnostic marker for the early detection of lung cancer (48–50).

Another recent study by Liu et al. confirms that DNA damage triggers nuclear translocation of cGAS and leads to the recruitment of cGAS to the site of double-stranded breaks, suppressing homologous recombination DNA repair (HR) and increasing genomic instability and, consequently, tumorigenesis (51). This observation is further supported by a study by Jiang et al., which found that nuclear cGAS inhibits HR in a STING-independent manner (52). These findings suggest that nuclear cGAS is a tumor enhancer by modulating the DNA damage response and influencing genome stability, indicating potential implications for inhibitors that block the nuclear translocation of cGAS for cancer intervention. The above studies suggest that nuclear DNA sensors play critical roles in tumorigenesis and might be a valuable prognostic marker for malignancy development and patient survival.

**Figure 1:**
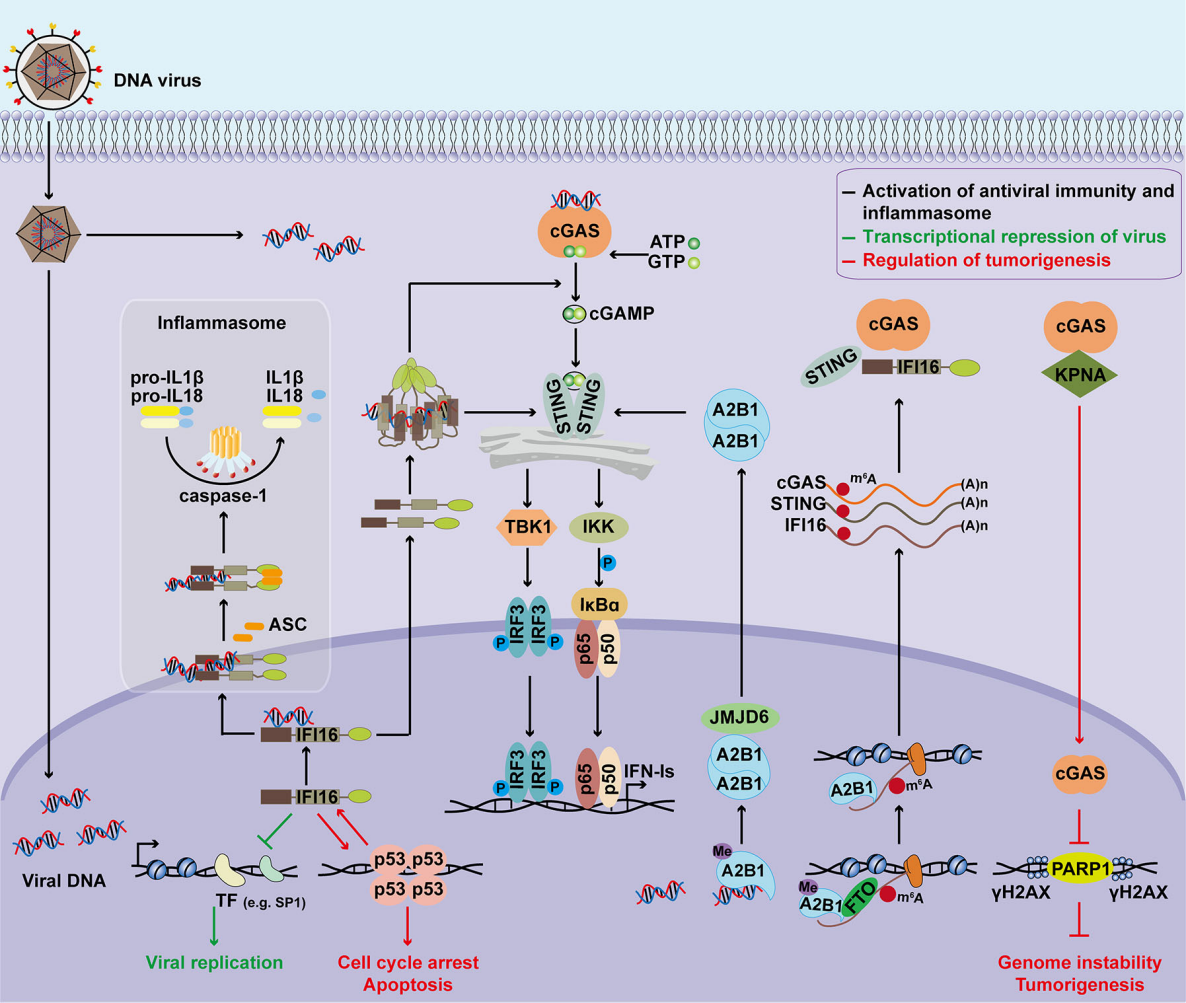
Major functions of nuclear DNA sensors. Upon detecting nuclear viral DNA, IFI16 is transported to the cytoplasm to activate the STING signaling cascade, inducing IFN‐Is expression through the TBK1‐IRF3 and NF‐κB axis. IFI16 also activates inflammasome to promote IL-1β and IL-18 maturation. Additionally, IFI16 functions as a transcriptional repressor to restrict viral replication by associating with transcription factors or promoters, preventing transcription factors from binding to promoters, and inducing chromatin marker changes. Nuclear hnRNPA2B1 dimerizes and is demethylated by JMJD6 after binding to viral dsDNA, resulting in the cytoplasmic translocation of hnRNPA2B1. The cytoplasmic hnRNPA2B1 activates the STING-TBK1-IRF3 signal to facilitate the transcription of IFN-Is. Moreover, demethylated hnRNPA2B1 enhances nucleocytoplasmic trafficking and translates cGAS, STING, and IFI16 mRNAs to amplify the antiviral immune response. Besides, the roles of nuclear DNA sensors during tumorigenesis have also been investigated. IFI16 is shown to act as a tumor suppressor in several types of cancers by interacting with p53 and enhancing p53-mediated transcriptional activation. In turn, functional activation of p53 stimulate the transcription of IFI16 through associating with the regulatory region of the *IFI16* promoter. DNA damage triggers nuclear translocation of cGAS. Nuclear cGAS promotes tumorigenesis by modulating the DNA damage response and increasing genomic instability. ASC, apoptosis-associated speck-like protein containing a CARD; STING, stimulator of interferon genes; TBK1, TANK-binding kinase 1; IKK, IκB kinase; IRF3, interferon regulatory factor 3; IFN-I, type I interferon; A2B1, hnRNPA2B1; JMJD6, jumonji domain containing 6; Me, methylation; m^6^A, *N^6^*-Methyladenosine; FTO, fat mass and obesity-associated protein; KPNA, karyopherin alpha; PARP1, Poly (ADP-Ribose) Polymerase 1. γH2AX, phosphorylated H2A histone family member X (H2AX) on serine 139.

**Figure 2:**
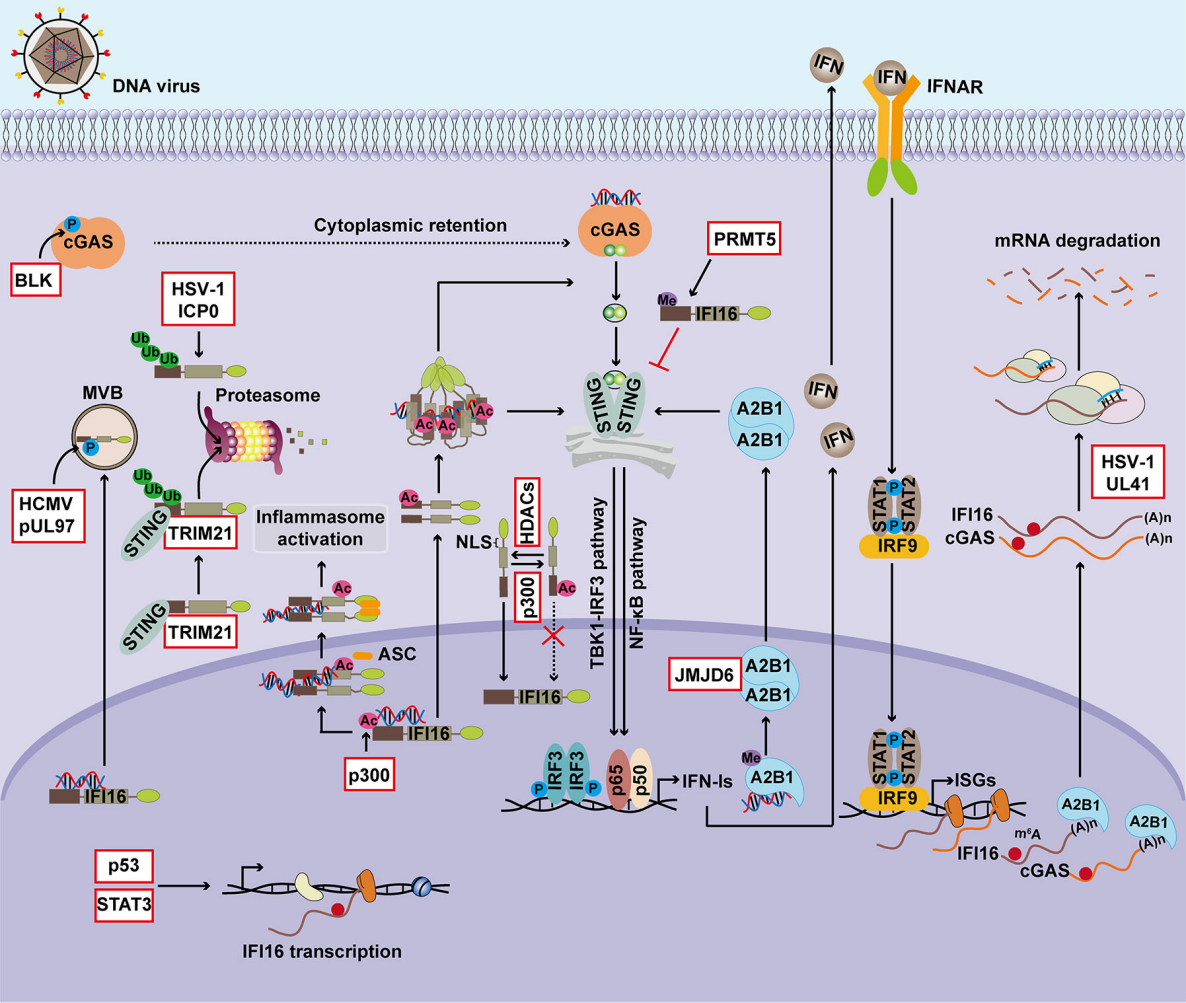
Regulation of nuclear DNA sensors. The expression and activation of DNA sensors are finely controlled during viral infection and tumorigenesis. p53 facilitates IFI16 transcription by directly binding to the promoter region of IFI16, and IL-6 drives IFI16 transcription in a STAT3-dependent manner. Post-transcriptional regulation also involves modulating the expression of nuclear DNA sensors. During DNA virus infection, hnRNPA2B1 functions as an m^6^A modulator to promote nucleocytoplasmic trafficking of cGAS and IFI16 mRNAs. UL41 from HSV-1 significantly reduces the expression of cGAS and IFI16 by degrading their transcripts. Furthermore, PTMs, particularly the phosphorylation, ubiquitination, acetylation, and methylation, play critical roles in regulating the activity and stability of nuclear DNA sensors. Phosphorylation of IFI16 controls its subcellular localization, and related antiviral immunity and BLK-mediated phosphorylation of cGAS facilitates its cytosolic retention. ICP0 from HSV-1 induces the ubiquitination and proteasome-dependent degradation of IFI16 and thus suppresses inflammasome activation. STING promotes IFI16 degradation via the ubiquitin-proteasome system by TRIM21. The acetyltransferase p300 mediates acetylation of IFI16 during HSV-1 infection, an essential step for inflammasome assembly and cytoplasmic translocation, activation of cytoplasmic STING signaling, and downstream IFN-β production. The sensing ability of IFI16 is modulated by acetylation of Lys99 and Lys128 within its NLS, and this PTM of IFI16 promotes the cytoplasmic translocation of IFI16, whereas HDACs promotes its nuclear import. hnRNPA2B1 is demethylated by JMJD6 in the HSV-1-infected cells, which consequently initiates IFN-α/β production and enhances STING-dependent cytoplasmic antiviral signaling. BLK, B-lymphoid tyrosine kinase; MVB, multivesicular bodies; ICP0, human HSV-1 infected cell polypeptide 0; TRIM21, tripartite motif-containing protein 21; Ub, Ubiquitination; Ac, Acetylation; Me, Methylation; STAT3, signal transducer and activator of transcription 3; NLS, nuclear localization signal; HDAC, histone deacetylase; PRMT5, protein arginine N-methyltransferase 5.

The functions of nuclear DNA sensors in regulating antiviral immunity, inflammasome activation, transcriptional repression, and tumorigenesis are summarized in **Figure 1**. Due to their important functions, the expression and cellular location of nuclear DNA sensors are tightly regulated (**Figure 2**).

## Transcriptional Regulation of Nuclear DNA Sensors

IFI16 mRNA is induced by both IFN-I (IFN-α and IFN-β) and IFN-II (IFN-γ) in multiple human cell lines such as human myeloid leukemia cells and fibrosarcoma cells (53, 54). IFNs are key molecules that contribute to the pathogenesis of systemic lupus erythematosus (SLE), and overproduction of IFN-I is always observed in patients with SLE (55–57). Consistently, the IFI16 transcripts in peripheral blood monocytes (PBMCs) of patients with SLE are significantly higher than that of healthy people (58). Infection with DNA viruses such as vaccinia virus (VACV), HSV-1, and human T-lymphotropic virus type 1 (HTLV-1) induces IFI16 expression dramatically (15, 20, 59, 60). IFI16 mRNA expression is correlated with high viral load and low CD4^+^T cell counts in HIV patients (61). IFI16 epigenetically suppresses hepatitis B virus (HBV) covalently closed circular DNA (cccDNA) by targeting an interferon-sensitive response element (ISRE) located in cccDNA. However, HBV infection downregulates the mRNA expression of IFI16 in the hepatocytes and liver tissues of patients with chronic hepatitis B (62). In addition to being tightly controlled transcriptionally during viral infection, IFI16 expression is precisely regulated during tumorigenesis. For instance, as mentioned above, IFI16 directly binds to the C-terminal region of p53 and enhances p53-mediated transcriptional activation (37, 38). Moreover, p53 also facilitates IFI16 transcription by directly binding to the promoter region of IFI16 and thus provides positive feedback regulation of p53 signaling (41). The IL-6/JAK/STAT3 pathway plays a key role in the growth and development of many human cancers (63). IL-6 treatment induces STAT3 phosphorylation and drives IFI16 transcription in a STAT3-dependent manner in human adenocarcinoma cell lines (64). The oncogene ZNF217 acts as a transcriptional repressor and plays an important role during neoplastic transformation (65–67). Consistent with its oncogenic role, ZNF217 represses the transcription of IFI16 (68).

hnRNPA2B1 mRNA levels are constitutively expressed during viral infection (20). By contrast, it is overexpressed in various malignant tumor tissues and cancer cell lines (69–72). For example, increased mRNA level of hnRNPA2B1 has been found in breast cancer cell lines deficient for breast cancer susceptibility gene 1 (BRCA1) expression. The restoration of BRCA1 expression reverts hnRNPA2B1 upregulation, implying the involvement of BRCA1 in the regulation of hnRNPA2B1 (73). Long non-coding RNA (lncRNA) CACNA1G-AS1 promotes the expression of hnRNPA2B1 in non-small cell lung cancer (NSCLC) cell lines, inducing malignant cell invasion, migration, and epithelial-mesenchymal transformation (EMT) (74). cGAS is an interferon-stimulated gene (ISG), and two adjacent ISREs in the promoter region of cGAS mediate the induction of cGAS by IFN-Is (75). The cGAS mRNA is upregulated in the PBMCs from patients with SLE (76). Both HSV-1 infection and IFN-α treatment induce cGAS mRNA expression in neonatal PBMCs from 1-month-old infants (77). LncRNA NEAT1 epigenetically inhibits cGAS expression to regulate the malignant phenotype of cancer cells and cytotoxic T cell infiltration in lung cancer (78).

## Post-Transcriptional Regulation of Nuclear DNA Sensors

Post-transcriptional regulation also plays a key role in modulating the expression of nuclear DNA sensors and related host antiviral immunity and tumorigenesis. Three isoforms of IFI16, isoform-A, B, and C, are widely detected in multiple cell lines and primary cells due to IFI16 pre-mRNA alternative splicing (79, 80). The spliceosome-associated factor, CTNNBL1, regulates the expression and alternative splicing of IFI16 and promotes proliferation and invasion in ovarian cancer (81). A novel transcript isoform of IFI16, which contains two HIN domains but lacks the PYD domain, interacts with AIM2 to impede the formation of a functional AIM2-ASC complex and inhibits AIM2 inflammasome (82). hnRNPA2B1 is a nuclear m^6^A reader and mediates m^6^A-dependent primary microRNA processing events (83). During DNA virus infection, hnRNPA2B1 functions as an m^6^A modulator to promote the m^6^A modification and nucleocytoplasmic trafficking of cGAS and IFI16 mRNAs after viral DNA recognition by hnRNPA2B1 (20). In addition to alternative splicing and m^6^A modification, viral proteins control IFI16 and cGAS mRNAs stability. UL41 from HSV-1 significantly degrades cGAS mRNA in HSV-1-infected human foreskin fibroblast (HFF) cells abrogating cGAS-STING-mediated IFN-I production dependent on its RNase activity (84). UL41 also reduces the expression of IFI16 by degrading its transcripts (85).

## Post-Translational Modification of Nuclear DNA Sensors

Post-translational modifications (PTMs) play important roles in regulating the activity, stability, and folding of targeted proteins by inducing their covalent linkage to new functional groups, such as phosphate, methyl group, and acetate (86). PTMs including phosphorylation, ubiquitination, methylation, and acetylation have been shown to influence PRR-dependent antiviral immunity and inflammatory responses by targeting the innate sensors and downstream signaling molecules, including receptors, adaptors, enzymes, and transcription factors (1, 86, 87). Moreover, PTMs dynamically change the compartmentalization, trafficking, and physical interaction of key molecules that control immunological processes. Here, this review summarizes the PTMs involved in the positive and negative regulation of the nuclear DNA sensors during viral infection and tumorigenesis, and a summary of the post-translational modifications of nuclear DNA sensors are listed in **Table 1**.

**Table 1:** PTMs of nuclear DNA sensors.

Target sensors	Regulators for PTM	PTMs	Mechanisms	References
IFI16	pUL97	Phosphorylation	pUL97 phosphorylates IFI16 during viral replication and re-localizes it from the nucleus to multivesicular bodies to overcome the restriction activity of IFI16	(88)
IFI16	ICP0	Ubiquitination	ICP0 promotes the ubiquitination and proteasome-dependent degradation of IFI16	(89)
IFI16	TRIM21	Ubiquitination	STING directly interacts with IFI16 and facilitates IFI16 ubiquitination and degradation via the ubiquitin-proteasome pathway by recruiting E3 ligase TRIM21	(90)
IFI16	p300	Acetylation	Acetylated IFI16 is essential for IFI16 cellular redistribution, inflammasome assembly in the cytoplasm, and activation of STING	(91, 92)
IFI16	HDACs	Deacetylation	HDACs activity promotes the nuclear import of IFI16	(91)
IFI16	PRMT5	Methylation	Methylated IFI16 suppresses dsDNA activation of STING pathways and attenuates IFN-I expression	(93)
cGAS	BLK	Phosphorylation	Phosphorylation of cGAS at Tyr205 by BLK facilitates its cytosolic retention	(51)
hnRNPA2B1	JMJD6	Demethylation	hnRNPA2B1 is demethylated by JMJD6 in the HSV-1-infected cells, which initiates IFN-I production	(20)

Phosphorylation is the most extensively investigated PTM type in antiviral innate immunity (87, 94). IFI16 contains a CcN motif that targets a heterologous protein to the nucleus and subsequently undergoes phosphorylation, particularly by the CcN-motif-phosphorylating protein kinase (CK2). The IFI16 CK2 phosphorylation site enhances nuclear import by facilitating binding to a nuclear component, and the nuclear-import characteristics of the IFI16 CcN motif were consistent with those of the HIV-1 Tat nuclear target signal (95). The viral protein kinase pUL97 of HCMV, which binds and phosphorylates nuclear IFI16, contributes to the nucleocytoplasmic translocation of IFI16 to overcome the restriction activity of IFI16 (88). These studies indicate that the phosphorylation of IFI16 controls IFI16 cellular location and relates antiviral immunity. Up until recently, there was no direct evidence indicating that IFI16 can be phosphorylated at specific sites upon inflammasome assembly.

Although IFI16 is required for the maximal phosphorylation and activation of p53 induced by ionizing radiation (38), it is unclear whether phosphorylation of IFI16 is also critical for its pro-apoptosis and antitumor role during tumorigenesis. DNA damage induces the nuclear translocation of cGAS, which suppresses DNA repair and promotes tumorigenesis by interacting with PARP (51). However, the B-lymphoid tyrosine kinase (BLK)-mediated phosphorylation of cGAS at Tyr215 facilitates the cytosolic retention of cGAS, which may be important for its antiviral role as a cytosolic DNA sensor (51).

Ubiquitination is also a key regulatory mechanism for nuclear DNA sensors, particularly for IFI16. The protein ubiquitination of target substrates involves a stepwise catalyzation by three enzymes, ubiquitin-activating enzyme (E1), ubiquitin-conjugating enzyme (E2), and ubiquitin ligase (E3) (96, 97), resulting in mono-ubiquitination. Ubiquitin can be further conjugated to additional ubiquitin moieties via the same three-step process, yielding polyubiquitin chains. Ubiquitin undergoes ubiquitination itself at its seven lysine residues (K6/K11/K27/K29/K33/K48/K63) or its amino-terminal methionine, which generates different types of ubiquitin chains with distinct functions (98). For instance, the K48-linked ubiquitin chain often induces the proteasomal degradation of targeted proteins, while the K63-linked ubiquitin chain is involved in the transduction of signaling pathways (99, 100). In addition to inducing IFN-I production as a DNA sensor, IFI16 induces the assembly of inflammasome complexes in response to DNA viruses, which is essential in immune protection against viral infections (17, 101). To counter IFI16-triggered antiviral immune responses, HSV-1 expresses an immediate-early protein, infected cell protein 0 (ICP0), an E3 ubiquitin ligase. After HSV-1 infection, ICP0 promotes the ubiquitination and proteasome-dependent degradation of IFI16 and suppresses inflammasome activation (89). Moreover, a previous study showed that the viral ICP0 protein leads to nuclear re-localization and the degradation of IFI16, resulting in the downstream inhibition of IRF3 signaling during HSV-1 infection (102). However, another study indicates that ICP0 is neither sufficient nor necessary for the degradation of IFI16 during HSV-1 infection (103). Due to these controversial results, the role of IFI16 ubiquitination mediated by ICP0 in antiviral immunity needs to be further clarified. Furthermore, it was recently found that STING facilitates ubiquitination on the first three lysines in the N-terminal region of IFI16 and promotes IFI16 degradation via the ubiquitin-proteasome pathway by recruiting the ubiquitin E3 ligase TRIM21 and restricting IFN-I overproduction during host antiviral immunity (90).

The acetylation of lysine residues, which is inversely regulated by acetyltransferases and deacetylases, occurs commonly in the proteome and plays an important role in numerous biological processes, such as chromatin remodeling, nuclear transport, and innate immunity (104). The sensing ability of IFI16 is modulated by acetylation of Lys99 and Lys128 within its nuclear localization signal (NLS), and the PTM of IFI16 promotes the translocation of IFI16 from the nucleus to the cytoplasm, whereas histone deacetylases (HDACs) promotes its nuclear import (91). The acetyltransferase p300 mediates acetylation of IFI16 during HSV-1 infection, which is essential for IFI16-inflammasome assembly in the nucleus and cytoplasmic translocation, activation of STING in the cytoplasm, and IFN-β production (92). Another relevant study also reported that IFI16 in complex with BRCA1-H2B or with BRCA1 recognizes the viral genome, leading to BRCA1 mediated p300 recruitment, interaction with IFI16, acetylation of IFI16 and H2B by p300, and the cytoplasmic transport of acetylated IFI16-H2B-BRCA1 via Ran GTP during KSHV or HSV-1 infection (105).

The methylation of lysine or arginine residues, which is inversely regulated by methyltransferases and demethylases, plays an important role in innate immune responses (106). A recent study demonstrated that IFI16 is methylated by protein arginine methyltransferase 5 (PRMT5) and suppresses the activation of the STING pathway (93). Moreover, a newly identified nuclear DNA sensor, hnRNPA2B1, is methylated in the resting cells. However, hnRNPA2B1 is demethylated by JMJD6 in the HSV-1-infected cells. Demethylated hnRNPA2B1 initiates IFN-α/β production and enhances STING-dependent cytoplasmic antiviral signaling (20).

Together, PTMs, particularly the phosphorylation, ubiquitination, acetylation, and methylation of nuclear DNA sensors, play a vital role in controlling antiviral immunity and tumorigenesis.

## Conclusion and Future Perspectives

Despite rapid advances in understanding of the functions and mechanisms of cytosolic DNA sensors in regulating host antiviral and antitumor immunity, studies that identify novel key nuclear DNA sensors and elucidate these functions are only just emerging. Given the important roles of nuclear DNA sensors during viral infection and tumorigenesis, it is critical to control expression. In this review, we briefly describe the nuclear functions of IFI16, hnRNPA2B1, and cGAS, and summarize the transcriptional, post-transcriptional, and post-translational regulation of these nuclear DNA sensors. However, several intriguing and important topics require further investigation.

The cytosolic DNA sensor, cGAS, has been found to translocate to the nucleus and is recruited to chromatin double-stranded breaks after DNA damage, where it suppresses homologous-recombination-mediated repair and promotes tumor growth (51, 52). Similarly, DNA-dependent protein kinase (DNA-PK) plays a critical role in the nucleus, where it is necessary for non-homologous end joining (NHEJ) and repairing double-strand DNA breaks. DNA-PK was recently identified as a cytosolic DNA sensor that activates a STING-independent DNA sensing pathway (107–109). These studies show that DNA-PK functions as a DNA sensor in the cytoplasm. However, considering that it predominantly localizes in the nucleus, it may also sense viral DNA in the nucleus and trigger an antiviral immune response like that of nuclear DNA sensors. Since all the three nuclear DNA sensors IFI16, hnRNPA2B1, and cGAS shuttle between cytoplasm and nucleus, all of them are involved in regulating both IFN-I-dependent antiviral immunity and tumorigenesis and newly identified nuclear DNA sensors may possess functions both in cytoplasm and nucleus.

The presence of host self-DNA generally in the nucleus was believed to be an immune-privileged cellular compartment. It is essential to understand how nuclear DNA sensors escape self-DNA-triggered activation in the immune response. cGAS has been reported to maintain an inhibitory state in the nucleus by binding nucleosome tighter to prevent autoreactivity to self-DNA (27, 110–112). A circular RNA named cia-cGAS has been identified to suppress nuclear cGAS by blocking its enzymatic activity, thereby preventing cGAS from sensing self-DNA to maintain host homeostasis (113). The multiple layers of regulation of nuclear DNA sensors may participate in avoiding inappropriate sensing self-DNA.

Invaded HSV-1 regulates IFI16 at multiple levels. HSV-1 infection-triggered IFN-I production induces IFI16 transcription (15, 20, 59, 60). UL41 protein from HSV-1 degrades IFI16 mRNA via its RNase activity and suppresses IFI16 expression post-transcriptionally (85). The ICP0 protein of HSV-1 degrades IFI16 post-translationally (89). HBV infection also downregulates the IFI16 mRNA level, which is worthy of further investigation (62). Several models have been proposed for the HBV-mediated inhibition of IFI16 expression: 1) HBV may suppress IFI16 transcription by promoting hypermethylation of IFI16 promoters; 2) HBV may stimulate the production of some non-coding RNAs to directly degrade the IFI16 mRNA or target the cellular factors responsible for IFI16 transcription; 3) HBV may actively suppress some innate immune signaling, which is important for IFI16 expression (62). There is always a race between host antiviral innate immunity and the immune evasion strategies of viruses (114). The novel regulation mechanism of nuclear DNA sensors by viral components will be an interesting focus in future studies.

PTMs, including phosphorylation, ubiquitination, methylation, and acetylation, have been shown to regulate the expression and activity of nuclear DNA sensors. Other PTMs, such as glutamylation, SUMOylation, and lactylation, also potentially regulate nuclear DNA sensors during antiviral immunity and tumorigenesis. Hence, three main aspects should be extensively investigated in the next few years: 1) the identification of more important nuclear DNA sensors; 2) elucidation of the novel strategies used by invaded viruses to inhibit the expression and function of nuclear DNA sensors; and, 3) the observation of more PTMs of nuclear DNA sensors and elucidation of related mechanisms.

## Author Contributions

FM conceived the idea. FZ, YY, and FM drafted the manuscript and created the figures. FM revised the manuscript and approved the submitted version.

## Funding

This work was supported by grants from the National Key Research and Development Program of China (2018YFA0900803), the National Natural Science Foundation of China (31670883, 31870912, and 82002222), and the Natural Science Foundation of Jiangsu Province (BK20200004).

## Conflict of Interest

The authors declare that the research was conducted in the absence of any commercial or financial relationships that could be construed as a potential conflict of interest.

## References

[1] Cao X Self-regulation and cross-regulation of pattern-recognition receptor signalling in health and disease. Nat Rev Immunol (2016) 16(1):35–50. 10.1038/nri.2015.8 26711677

[2] Ishikawa H Barber GN STING is an endoplasmic reticulum adaptor that facilitates innate immune signalling. Nature (2008) 455(7213):674–8. 10.1038/nature07317 PMC2804933 18724357

[3] Chen Q Sun L Chen ZJ Regulation and function of the cGAS-STING pathway of cytosolic DNA sensing. Nat Immunol (2016) 17(10):1142–9. 10.1038/ni.3558 27648547

[4] Galluzzi L Vanpouille-Box C Bakhoum SF Demaria S SnapShot: CGAS-STING Signaling. Cell (2018) 173(1):276–276.e1. 10.1016/j.cell.2018.03.015 29570996

[5] Fitzgerald KA McWhirter SM Faia KL Rowe DC Latz E Golenbock DT IKKepsilon and TBK1 are essential components of the IRF3 signaling pathway. Nat Immunol (2003) 4(5):491–6. 10.1038/ni921 12692549

[6] Hayden MS Ghosh S Shared principles in NF-kappaB signaling. Cell (2008) 132(3):344–62. 10.1016/j.cell.2008.01.020 18267068

[7] Sato M Suemori H Hata N Asagiri M Ogasawara K Nakao K Distinct and essential roles of transcription factors IRF-3 and IRF-7 in response to viruses for IFN-alpha/beta gene induction. Immunity (2000) 13(4):539–48. 10.1016/s1074-7613(00)00053-4 11070172

[8] Evavold CL Kagan JC Inflammasomes: Threat-Assessment Organelles of the Innate Immune System. Immunity (2019) 51(4):609–24. 10.1016/j.immuni.2019.08.005 PMC6801093 31473100

[9] Wang L Sun L Byrd KM Ko CC Zhao Z Fang J AIM2 Inflammasome’s First Decade of Discovery: Focus on Oral Diseases. Front Immunol (2020) 11:1487. 10.3389/fimmu.2020.01487 32903550 PMC7438472

[10] Diner BA Lum KK Cristea IM The emerging role of nuclear viral DNA sensors. J Biol Chem (2015) 290(44):26412–21. 10.1074/jbc.R115.652289 PMC4646299 26354430

[11] Komatsu T Nagata K Wodrich H The Role of Nuclear Antiviral Factors against Invading DNA Viruses: The Immediate Fate of Incoming Viral Genomes. Viruses (2016) 8(10):290. 10.3390/v8100290 PMC5086622 27782081

[12] Zhang X Flavell RA Li HB hnRNPA2B1: a nuclear DNA sensor in antiviral immunity. Cell Res (2019) 29(11):879–80. 10.1038/s41422-019-0226-8 PMC6889404 31471560

[13] Lum KK Howard TR Pan C Cristea IM Charge-Mediated Pyrin Oligomerization Nucleates Antiviral IFI16 Sensing of Herpesvirus DNA. mBio (2019) 10(4):e01428-19. 10.1128/mBio.01428-19 31337724 PMC6650555

[14] Chan YK Gack MU Viral evasion of intracellular DNA and RNA sensing. Nat Rev Microbiol (2016) 14(6):360–73. 10.1038/nrmicro.2016.45 PMC5072394 27174148

[15] Unterholzner L Keating SE Baran M Horan KA Jensen SB Sharma S IFI16 is an innate immune sensor for intracellular DNA. Nat Immunol (2010) 11(11):997–1004. 10.1038/ni.1932 20890285 PMC3142795

[16] Almine JF O’Hare CA Dunphy G Haga IR Naik RJ Atrih A IFI16 and cGAS cooperate in the activation of STING during DNA sensing in human keratinocytes. Nat Commun (2017) 8:14392. 10.1038/ncomms14392 28194029 PMC5316833

[17] Kerur N Veettil MV Sharma-Walia N Bottero V Sadagopan S Otageri P IFI16 acts as a nuclear pathogen sensor to induce the inflammasome in response to Kaposi Sarcoma-associated herpesvirus infection. Cell Host Microbe (2011) 9(5):363–75. 10.1016/j.chom.2011.04.008 PMC3113467 21575908

[18] Singh VV Kerur N Bottero V Dutta S Chakraborty S Ansari MA Kaposi’s sarcoma-associated herpesvirus latency in endothelial and B cells activates gamma interferon-inducible protein 16-mediated inflammasomes. J Virol (2013) 87(8):4417–31. 10.1128/JVI.03282-12 PMC3624349 23388709

[19] Brunette RL Young JM Whitley DG Brodsky IE Malik HS Stetson DB Extensive evolutionary and functional diversity among mammalian AIM2-like receptors. J Exp Med (2012) 209(11):1969–83. 10.1084/jem.20121960 PMC3478938 23045604

[20] Wang L Wen M Cao X Nuclear hnRNPA2B1 initiates and amplifies the innate immune response to DNA viruses. Science (2019) 365(6454):eaav0758. 10.1126/science.aav0758 31320558

[21] Zhou X Wang L Zou W Chen X Roizman B Zhou GG hnRNPA2B1 Associated with Recruitment of RNA into Exosomes Plays a Key Role in Herpes Simplex Virus 1 Release from Infected Cells. J Virol (2020) 94(13):e00367-20. 10.1128/jvi.00367-20 32295924 PMC7307161

[22] Pingale KD Kanade GD Karpe YA Heterogeneous Nuclear Ribonucleoproteins Participate in Hepatitis E Virus Replication. J Mol Biol (2020) 432(7):2369–87. 10.1016/j.jmb.2020.02.025 32119874

[23] Sun L Wu J Du F Chen X Chen ZJ Cyclic GMP-AMP synthase is a cytosolic DNA sensor that activates the type I interferon pathway. Science (2013) 339(6121):786–91. 10.1126/science.1232458 PMC3863629 23258413

[24] Zhong L Hu MM Bian LJ Liu Y Chen Q Shu HB Phosphorylation of cGAS by CDK1 impairs self-DNA sensing in mitosis. Cell Discovery (2020) 6:26. 10.1038/s41421-020-0162-2 32351706 PMC7186227

[25] Denais CM Gilbert RM Isermann P McGregor AL te Lindert M Weigelin B Nuclear envelope rupture and repair during cancer cell migration. Science (2016) 352(6283):353–8. 10.1126/science.aad7297 PMC4833568 27013428

[26] Zhao B Xu P Rowlett CM Jing T Shinde O Lei Y The Molecular Basis of Tight Nuclear Tethering and Inactivation of cGAS. Nature (2020) 587(7835):673–7. 10.1038/s41586-020-2749-z PMC7704945 32911481

[27] Kujirai T Zierhut C Takizawa Y Kim R Negishi L Uruma N Structural basis for the inhibition of cGAS by nucleosomes. Science (2020) 370(6515):455–8. 10.1126/science.abd0237 PMC7584773 32912999

[28] Lahaye X Gentili M Silvin A Conrad C Picard L Jouve M NONO Detects the Nuclear HIV Capsid to Promote cGAS-Mediated Innate Immune Activation. Cell (2018) 175(2):488–501.e22. 10.1016/j.cell.2018.08.062 30270045

[29] Johnstone RW Kerry JA Trapani JA The human interferon-inducible protein, IFI 16, is a repressor of transcription. J Biol Chem (1998) 273(27):17172–7. 10.1074/jbc.273.27.17172 9642285

[30] Gariano GR Dell’Oste V Bronzini M Gatti D Luganini A De Andrea M The intracellular DNA sensor IFI16 gene acts as restriction factor for human cytomegalovirus replication. PloS Pathog (2012) 8(1):e1002498. 10.1371/journal.ppat.1002498 22291595 PMC3266931

[31] Johnson KE Bottero V Flaherty S Dutta S Singh VV Chandran B IFI16 restricts HSV-1 replication by accumulating on the hsv-1 genome, repressing HSV-1 gene expression, and directly or indirectly modulating histone modifications. PloS Pathog (2014) 10(11):e1004503. 10.1371/journal.ppat.1004503 25375629 PMC4223080

[32] Diner BA Lum KK Toettcher JE Cristea IM Viral DNA Sensors IFI16 and Cyclic GMP-AMP Synthase Possess Distinct Functions in Regulating Viral Gene Expression, Immune Defenses, and Apoptotic Responses during Herpesvirus Infection. mBio (2016) 7(6):e01553-16. 10.1128/mBio.01553-16 27935834 PMC5111403

[33] Lo Cigno I De Andrea M Borgogna C Albertini S Landini MM Peretti A The Nuclear DNA Sensor IFI16 Acts as a Restriction Factor for Human Papillomavirus Replication through Epigenetic Modifications of the Viral Promoters. J Virol (2015) 89(15):7506–20. 10.1128/JVI.00013-15 PMC4505635 25972554

[34] Hotter D Bosso M Jonsson KL Krapp C Sturzel CM Das A IFI16 Targets the Transcription Factor Sp1 to Suppress HIV-1 Transcription and Latency Reactivation. Cell Host Microbe (2019) 25(6):858–72.e13. 10.1016/j.chom.2019.05.002 31175045 PMC6681451

[35] Cristea IM Moorman NJ Terhune SS Cuevas CD O’Keefe ES Rout MP Human cytomegalovirus pUL83 stimulates activity of the viral immediate-early promoter through its interaction with the cellular IFI16 protein. J Virol (2010) 84(15):7803–14. 10.1128/JVI.00139-10 PMC2897612 20504932

[36] Orzalli MH Conwell SE Berrios C DeCaprio JA Knipe DM Nuclear interferon-inducible protein 16 promotes silencing of herpesviral and transfected DNA. Proc Natl Acad Sci USA (2013) 110(47):E4492–501. 10.1073/pnas.1316194110 PMC3839728 24198334

[37] Choubey D Panchanathan R IFI16, an amplifier of DNA-damage response: Role in cellular senescence and aging-associated inflammatory diseases. Ageing Res Rev (2016) 28:27–36. 10.1016/j.arr.2016.04.002 27063514

[38] Fujiuchi N Aglipay JA Ohtsuka T Maehara N Sahin F Su GH Requirement of IFI16 for the maximal activation of p53 induced by ionizing radiation. J Biol Chem (2004) 279(19):20339–44. 10.1074/jbc.M400344200 14990579

[39] De Andrea M Gioia D Mondini M Azzimonti B Reno F Pecorari G Effects of IFI16 overexpression on the growth and doxorubicin sensitivity of head and neck squamous cell carcinoma-derived cell lines. Head Neck (2007) 29(9):835–44. 10.1002/hed.20611 17510972

[40] Lin W Zhao Z Ni Z Zhao Y Du W Chen S IFI16 restoration in hepatocellular carcinoma induces tumour inhibition via activation of p53 signals and inflammasome. Cell Prolif (2017) 50(6):e12392. 10.1111/cpr.12392 PMC6529087 28990231

[41] Song LL Alimirah F Panchanathan R Xin H Choubey D Expression of an IFN-inducible cellular senescence gene, IFI16, is up-regulated by p53. Mol Cancer Res (2008) 6(11):1732–41. 10.1158/1541-7786.Mcr-08-0208 18974396

[42] Cai H Yan L Liu N Xu M Cai H IFI16 promotes cervical cancer progression by upregulating PD-L1 in immunomicroenvironment through STING-TBK1-NF-kB pathway. BioMed Pharmacother (2020) 123:109790. 10.1016/j.biopha.2019.109790 31896065

[43] Golan-Gerstl R Cohen M Shilo A Suh SS Bakàcs A Coppola L Splicing factor hnRNP A2/B1 regulates tumor suppressor gene splicing and is an oncogenic driver in glioblastoma. Cancer Res (2011) 71(13):4464–72. 10.1158/0008-5472.Can-10-4410 21586613

[44] Dai S Zhang J Huang S Lou B Fang B Ye T HNRNPA2B1 regulates the epithelial-mesenchymal transition in pancreatic cancer cells through the ERK/snail signalling pathway. Cancer Cell Int (2017) 17:12. 10.1186/s12935-016-0368-4 28077929 PMC5223355

[45] Chen T Gu C Xue C Yang T Zhong Y Liu S LncRNA-uc002mbe.2 Interacting with hnRNPA2B1 Mediates AKT Deactivation and p21 Up-Regulation Induced by Trichostatin in Liver Cancer Cells. Front Pharmacol (2017) 8:669. 10.3389/fphar.2017.00669 28993733 PMC5622184

[46] Barceló C Etchin J Mansour MR Sanda T Ginesta MM Sanchez-Arévalo Lobo VJ Ribonucleoprotein HNRNPA2B1 interacts with and regulates oncogenic KRAS in pancreatic ductal adenocarcinoma cells. Gastroenterology (2014) 147(4):882–92.e8. 10.1053/j.gastro.2014.06.041 24998203

[47] Yang Y Wei Q Tang Y Yuanyuan W Luo Q Zhao H Loss of hnRNPA2B1 inhibits malignant capability and promotes apoptosis via down-regulating Lin28B expression in ovarian cancer. Cancer Lett (2020) 475:43–52. 10.1016/j.canlet.2020.01.029 32006618

[48] Zhou J Mulshine JL Unsworth EJ Scott FM Avis IM Vos MD Purification and characterization of a protein that permits early detection of lung cancer. Identification of heterogeneous nuclear ribonucleoprotein-A2/B1 as the antigen for monoclonal antibody 703D4. J Biol Chem (1996) 271(18):10760–6. 10.1074/jbc.271.18.10760 8631886

[49] Fielding P Turnbull L Prime W Walshaw M Field JK Heterogeneous nuclear ribonucleoprotein A2/B1 up-regulation in bronchial lavage specimens: a clinical marker of early lung cancer detection. Clin Cancer Res (1999) 5(12):4048–52. 10632338

[50] Tauler J Zudaire E Liu H Shih J Mulshine JL hnRNP A2/B1 modulates epithelial-mesenchymal transition in lung cancer cell lines. Cancer Res (2010) 70(18):7137–47. 10.1158/0008-5472.CAN-10-0860 20807810

[51] Liu H Zhang H Wu X Ma D Wu J Wang L Nuclear cGAS suppresses DNA repair and promotes tumorigenesis. Nature (2018) 563(7729):131–6. 10.1038/s41586-018-0629-6 30356214

[52] Jiang H Xue X Panda S Kawale A Hooy RM Liang F Chromatin-bound cGAS is an inhibitor of DNA repair and hence accelerates genome destabilization and cell death. EMBO J (2019) 38(21):e102718. 10.15252/embj.2019102718 31544964 PMC6826206

[53] Dawson MJ Trapani JA IFI 16 gene encodes a nuclear protein whose expression is induced by interferons in human myeloid leukaemia cell lines. J Cell Biochem (1995) 57(1):39–51. 10.1002/jcb.240570106 7536752

[54] Der SD Zhou A Williams BR Silverman RH Identification of genes differentially regulated by interferon alpha, beta, or gamma using oligonucleotide arrays. Proc Natl Acad Sci USA (1998) 95(26):15623–8. 10.1073/pnas.95.26.15623 PMC28094 9861020

[55] Hooks JJ Moutsopoulos HM Geis SA Stahl N II Decker JL Notkins AL Immune interferon in the circulation of patients with autoimmune disease. N Engl J Med (1979) 301(1):5–8. 10.1056/NEJM197907053010102 449915

[56] Banchereau J Pascual V Type I interferon in systemic lupus erythematosus and other autoimmune diseases. Immunity (2006) 25(3):383–92. 10.1016/j.immuni.2006.08.010 16979570

[57] Oke V Gunnarsson I Dorschner J Eketjall S Zickert A Niewold TB High levels of circulating interferons type I, type II and type III associate with distinct clinical features of active systemic lupus erythematosus. Arthritis Res Ther (2019) 21(1):107. 10.1186/s13075-019-1878-y 31036046 PMC6489203

[58] Kimkong I Avihingsanon Y Hirankarn N Expression profile of HIN200 in leukocytes and renal biopsy of SLE patients by real-time RT-PCR. Lupus (2009) 18(12):1066–72. 10.1177/0961203309106699 19762380

[59] Thompson MR Sharma S Atianand M Jensen SB Carpenter S Knipe DM Interferon gamma-inducible protein (IFI) 16 transcriptionally regulates type i interferons and other interferon-stimulated genes and controls the interferon response to both DNA and RNA viruses. J Biol Chem (2014) 289(34):23568–81. 10.1074/jbc.M114.554147 PMC4156042 25002588

[60] Yang B Song D Liu Y Cui Y Lu G Di W IFI16 regulates HTLV-1 replication through promoting HTLV-1 RTI-induced innate immune responses. FEBS Lett (2018) 592(10):1693–704. 10.1002/1873-3468.13077 29710427

[61] Nissen SK Hojen JF Andersen KL Kofod-Olsen E Berg RK Paludan SR Innate DNA sensing is impaired in HIV patients and IFI16 expression correlates with chronic immune activation. Clin Exp Immunol (2014) 177(1):295–309. 10.1111/cei.12317 24593816 PMC4089180

[62] Yang Y Zhao X Wang Z Shu W Li L Li Y Nuclear Sensor Interferon-Inducible Protein 16 Inhibits the Function of Hepatitis B Virus Covalently Closed Circular DNA by Integrating Innate Immune Activation and Epigenetic Suppression. Hepatology (2020) 71(4):1154–69. 10.1002/hep.30897 31402464

[63] Johnson DE O’Keefe RA Grandis JR Targeting the IL-6/JAK/STAT3 signalling axis in cancer. Nat Rev Clin Oncol (2018) 15(4):234–48. 10.1038/nrclinonc.2018.8 PMC5858971 29405201

[64] Ni Z Bremner R Brahma-related gene 1-dependent STAT3 recruitment at IL-6-inducible genes. J Immunol (2007) 178(1):345–51. 10.4049/jimmunol.178.1.345 17182572

[65] Collins C Rommens JM Kowbel D Godfrey T Tanner M Hwang S II Positional cloning of ZNF217 and NABC1: genes amplified at 20q13.2 and overexpressed in breast carcinoma. Proc Natl Acad Sci USA (1998) 95(15):8703–8. 10.1073/pnas.95.15.8703 PMC21140 9671742

[66] Iwabuchi H Sakamoto M Sakunaga H Ma YY Carcangiu ML Pinkel D Genetic analysis of benign, low-grade, and high-grade ovarian tumors. Cancer Res (1995) 55(24):6172–80. 8521410

[67] Bar-Shira A Pinthus JH Rozovsky U Goldstein M Sellers WR Yaron Y Multiple genes in human 20q13 chromosomal region are involved in an advanced prostate cancer xenograft. Cancer Res (2002) 62(23):6803–7. 12460888

[68] Krig SR Jin VX Bieda MC O’Geen H Yaswen P Green R Identification of genes directly regulated by the oncogene ZNF217 using chromatin immunoprecipitation (ChIP)-chip assays. J Biol Chem (2007) 282(13):9703–12. 10.1074/jbc.M611752200 PMC2269729 17259635

[69] von Eckardstein KL Patt S Zhu J Zhang L Cervos-Navarro J Reszka R Short-term neuropathological aspects of in vivo suicide gene transfer to the F98 rat glioblastoma using liposomal and viral vectors. Histol Histopathol (2001) 16(3):735–44. 10.14670/HH-16.735 11510963

[70] Yan-Sanders Y Hammons GJ Lyn-Cook BD Increased expression of heterogeneous nuclear ribonucleoprotein A2/B1 (hnRNP) in pancreatic tissue from smokers and pancreatic tumor cells. Cancer Lett (2002) 183(2):215–20. 10.1016/s0304-3835(02)00168-4 12065097

[71] Lee CL Hsiao HH Lin CW Wu SP Huang SY Wu CY Strategic shotgun proteomics approach for efficient construction of an expression map of targeted protein families in hepatoma cell lines. Proteomics (2003) 3(12):2472–86. 10.1002/pmic.200300586 14673797

[72] Lee CH Lum JH Cheung BP Wong MS Butt YK Tam MF Identification of the heterogeneous nuclear ribonucleoprotein A2/B1 as the antigen for the gastrointestinal cancer specific monoclonal antibody MG7. Proteomics (2005) 5(4):1160–6. 10.1002/pmic.200401159 15759317

[73] Santarosa M Del Col L Viel A Bivi N D’Ambrosio C Scaloni A BRCA1 modulates the expression of hnRNPA2B1 and KHSRP. Cell Cycle (2010) 9(23):4666–73. 10.4161/cc.9.23.14022 PMC3048036 21099359

[74] Yu PF Kang AR Jing LJ Wang YM Long non-coding RNA CACNA1G-AS1 promotes cell migration, invasion and epithelial-mesenchymal transition by HNRNPA2B1 in non-small cell lung cancer. Eur Rev Med Pharmacol Sci (2018) 22(4):993–1002. 10.26355/eurrev_201802_14381 29509247

[75] Ma F Li B Liu SY Iyer SS Yu Y Wu A Positive feedback regulation of type I IFN production by the IFN-inducible DNA sensor cGAS. J Immunol (2015) 194(4):1545–54. 10.4049/jimmunol.1402066 PMC4324085 25609843

[76] An J Durcan L Karr RM Briggs TA Rice G II Teal TH Expression of Cyclic GMP-AMP Synthase in Patients With Systemic Lupus Erythematosus. Arthritis Rheumatol (2017) 69(4):800–7. 10.1002/art.40002 27863149

[77] Wang ZS Liu YL Mi N Duan DY Intracellular DNA sensing pathway of cGAS-cGAMP is decreased in human newborns and young children. Mol Immunol (2017) 87:76–85. 10.1016/j.molimm.2017.04.007 28412547

[78] Ma F Lei YY Ding MG Luo LH Xie YC Liu XL LncRNA NEAT1 Interacted With DNMT1 to Regulate Malignant Phenotype of Cancer Cell and Cytotoxic T Cell Infiltration via Epigenetic Inhibition of p53, cGAS, and STING in Lung Cancer. Front Genet (2020) 11:250. 10.3389/fgene.2020.00250 32296457 PMC7136539

[79] Johnstone RW Kershaw MH Trapani JA Isotypic variants of the interferon-inducible transcriptional repressor IFI 16 arise through differential mRNA splicing. Biochemistry (1998) 37(34):11924–31. 10.1021/bi981069a 9718316

[80] Veeranki S Choubey D Interferon-inducible p200-family protein IFI16, an innate immune sensor for cytosolic and nuclear double-stranded DNA: regulation of subcellular localization. Mol Immunol (2012) 49(4):567–71. 10.1016/j.molimm.2011.11.004 PMC3249514 22137500

[81] Li Y Guo H Jin C Qiu C Gao M Zhang L Spliceosome-associated factor CTNNBL1 promotes proliferation and invasion in ovarian cancer. Exp Cell Res (2017) 357(1):124–34. 10.1016/j.yexcr.2017.05.008 28501461

[82] Wang PH Ye ZW Deng JJ Siu KL Gao WW Chaudhary V Inhibition of AIM2 inflammasome activation by a novel transcript isoform of IFI16. EMBO Rep (2018) 19(10):e45737. 10.15252/embr.201845737 30104205 PMC6172465

[83] Alarcon CR Goodarzi H Lee H Liu X Tavazoie S Tavazoie SF HNRNPA2B1 Is a Mediator of m(6)A-Dependent Nuclear RNA Processing Events. Cell (2015) 162(6):1299–308. 10.1016/j.cell.2015.08.011 PMC4673968 26321680

[84] Su C Zheng C Herpes Simplex Virus 1 Abrogates the cGAS/STING-Mediated Cytosolic DNA-Sensing Pathway via Its Virion Host Shutoff Protein, UL41. J Virol (2017) 91(6):e02414-16. 10.1128/JVI.02414-16 28077645 PMC5331819

[85] Orzalli MH Broekema NM Knipe DM Relative Contributions of Herpes Simplex Virus 1 ICP0 and vhs to Loss of Cellular IFI16 Vary in Different Human Cell Types. J Virol (2016) 90(18):8351–9. 10.1128/JVI.00939-16 PMC5008076 27412599

[86] Deribe YL Pawson T Dikic I Post-translational modifications in signal integration. Nat Struct Mol Biol (2010) 17(6):666–72. 10.1038/nsmb.1842 20495563

[87] Liu J Qian C Cao X Post-Translational Modification Control of Innate Immunity. Immunity (2016) 45(1):15–30. 10.1016/j.immuni.2016.06.020 27438764

[88] Dell’Oste V Gatti D Gugliesi F De Andrea M Bawadekar M Lo Cigno I Innate nuclear sensor IFI16 translocates into the cytoplasm during the early stage of in vitro human cytomegalovirus infection and is entrapped in the egressing virions during the late stage. J Virol (2014) 88(12):6970–82. 10.1128/JVI.00384-14 PMC4054358 24696486

[89] Johnson KE Chikoti L Chandran B Herpes simplex virus 1 infection induces activation and subsequent inhibition of the IFI16 and NLRP3 inflammasomes. J Virol (2013) 87(9):5005–18. 10.1128/JVI.00082-13 PMC3624293 23427152

[90] Li D Wu R Guo W Xie L Qiao Z Chen S STING-Mediated IFI16 Degradation Negatively Controls Type I Interferon Production. Cell Rep (2019) 29(5):1249–60.e4. 10.1016/j.celrep.2019.09.069 31665637

[91] Li T Diner BA Chen J Cristea IM Acetylation modulates cellular distribution and DNA sensing ability of interferon-inducible protein IFI16. Proc Natl Acad Sci U.S.A. (2012) 109(26):10558–63. 10.1073/pnas.1203447109 PMC3387042 22691496

[92] Ansari MA Dutta S Veettil MV Dutta D Iqbal J Kumar B Herpesvirus Genome Recognition Induced Acetylation of Nuclear IFI16 Is Essential for Its Cytoplasmic Translocation, Inflammasome and IFN-beta Responses. PloS Pathog (2015) 11(7):e1005019. 10.1371/journal.ppat.1005019 26134128 PMC4489722

[93] Kim H Kim H Feng Y Li Y Tamiya H Tocci S PRMT5 control of cGAS/STING and NLRC5 pathways defines melanoma response to antitumor immunity. Sci Transl Med (2020) 12(551):eaaz5683. 10.1126/scitranslmed.aaz5683 32641491 PMC7508354

[94] Zhou Y He C Wang L Ge B Post-translational regulation of antiviral innate signaling. Eur J Immunol (2017) 47(9):1414–26. 10.1002/eji.201746959 PMC7163624 28744851

[95] Briggs LJ Johnstone RW Elliot RM Xiao CY Dawson M Trapani JA Novel properties of the protein kinase CK2-site-regulated nuclear- localization sequence of the interferon-induced nuclear factor IFI 16. Biochem J (2001) 353(Pt 1):69–77. 10.1042/bj3530069 11115400 PMC1221544

[96] Komander D Rape M The ubiquitin code. Annu Rev Biochem (2012) 81:203–29. 10.1146/annurev-biochem-060310-170328 22524316

[97] Swatek KN Komander D Ubiquitin modifications. Cell Res (2016) 26(4):399–422. 10.1038/cr.2016.39 27012465 PMC4822133

[98] Walczak H Iwai K Dikic I Generation and physiological roles of linear ubiquitin chains. BMC Biol (2012) 10:23. 10.1186/1741-7007-10-23 22420778 PMC3305636

[99] Jiang X Chen ZJ The role of ubiquitylation in immune defence and pathogen evasion. Nat Rev Immunol (2011) 12(1):35–48. 10.1038/nri3111 22158412 PMC3864900

[100] Heaton SM Borg NA Dixit VM Ubiquitin in the activation and attenuation of innate antiviral immunity. J Exp Med (2016) 213(1):1–13. 10.1084/jem.20151531 26712804 PMC4710203

[101] Ansari MA Singh VV Dutta S Veettil MV Dutta D Chikoti L Constitutive interferon-inducible protein 16-inflammasome activation during Epstein-Barr virus latency I, II, and III in B and epithelial cells. J Virol (2013) 87(15):8606–23. 10.1128/JVI.00805-13 PMC3719826 23720728

[102] Orzalli MH DeLuca NA Knipe DM Nuclear IFI16 induction of IRF-3 signaling during herpesviral infection and degradation of IFI16 by the viral ICP0 protein. (2012) Proc Natl Acad Sci U S A (2012) 109(44):E3008-17. 10.1073/pnas.1211302109 23027953 PMC3497734

[103] Cuchet-Lourenco D Anderson G Sloan E Orr A Everett RD The viral ubiquitin ligase ICP0 is neither sufficient nor necessary for degradation of the cellular DNA sensor IFI16 during herpes simplex virus 1 infection. J Virol (2013) 87(24):13422–32. 10.1128/JVI.02474-13 PMC3838218 24089555

[104] Choudhary C Kumar C Gnad F Nielsen ML Rehman M Walther TC Lysine acetylation targets protein complexes and co-regulates major cellular functions. Science (2009) 325(5942):834–40. 10.1126/science.1175371 19608861

[105] Iqbal J Ansari MA Kumar B Dutta D Roy A Chikoti L Histone H2B-IFI16 Recognition of Nuclear Herpesviral Genome Induces Cytoplasmic Interferon-beta Responses. PloS Pathog (2016) 12(10):e1005967. 10.1371/journal.ppat.1005967 27764250 PMC5072618

[106] Beaver JE Waters ML Molecular Recognition of Lys and Arg Methylation. ACS Chem Biol (2016) 11(3):643–53. 10.1021/acschembio.5b00996 26759915

[107] Lieber MR Ma Y Pannicke U Schwarz K Mechanism and regulation of human non-homologous DNA end-joining. Nat Rev Mol Cell Biol (2003) 4(9):712–20. 10.1038/nrm1202 14506474

[108] Ferguson BJ Mansur DS Peters NE Ren H Smith GL DNA-PK is a DNA sensor for IRF-3-dependent innate immunity. Elife (2012) 1:e00047. 10.7554/eLife.00047 23251783 PMC3524801

[109] Burleigh K Maltbaek JH Cambier S Green R Gale M Jr. James RC Human DNA-PK activates a STING-independent DNA sensing pathway. Sci Immunol (2020) 5(43):eaba4219. 10.1126/sciimmunol.aba4219 31980485 PMC7081723

[110] Michalski S de Oliveira Mann CC Stafford CA Witte G Bartho J Lammens K Structural basis for sequestration and autoinhibition of cGAS by chromatin. Nature (2020). 587(7835):678–82. 10.1038/s41586-020-2748-0 32911480

[111] Boyer JA Spangler CJ Strauss JD Cesmat AP Liu P McGinty RK Structural basis of nucleosome-dependent cGAS inhibition. Science (2020) 370(6515):450–4. 10.1126/science.abd0609 PMC8189757 32913000

[112] Pathare GR Decout A Glück S Cavadini S Makasheva K Hovius R Structural mechanism of cGAS inhibition by the nucleosome. Nature (2020) 587(7835):668–72. 10.1038/s41586-020-2750-6 32911482

[113] Xia P Wang S Ye B Du Y Li C Xiong Z A Circular RNA Protects Dormant Hematopoietic Stem Cells from DNA Sensor cGAS-Mediated Exhaustion. Immunity (2018) 48(4):688–701.e7. 10.1016/j.immuni.2018.03.016 29625897

[114] Zhu H Zheng C The Race between Host Antiviral Innate Immunity and the Immune Evasion Strategies of Herpes Simplex Virus 1. Microbiol Mol Biol Rev (2020) 84(4):e00099-20. 10.1128/MMBR.00099-20 32998978 PMC7528619

